# Perceptions of Nurse Managers and Supervisors on the Scope of Nursing Practice Following the 2023 National Regulatory Update in Saudi Arabia: A Qualitative Study in Makkah Cluster Hospitals

**DOI:** 10.3390/healthcare14142198

**Published:** 2026-07-20

**Authors:** Hanan M. Alfahmi, Ali Kerari

**Affiliations:** 1 Makkah Health Cluster, Makkah 24245, Saudi Arabia; hamalfahmi@moh.gov.sa; 2 Nursing Administration and Education Department, College of Nursing, King Saud University, Riyadh 11421, Saudi Arabia

**Keywords:** scope of nursing practice, qualitative research, thematic analysis, Makkah Health Cluster, Saudi Arabia, role ambiguity, Saudi Vision 2030, nursing regulation, professional autonomy

## Abstract

**Highlights:**

**What are the main findings?**

- Nurse managers and supervisors in Makkah Cluster Hospitals perceived the scope of nursing practice as being variably enacted and frequently constrained, with role ambiguity and administrative barriers persisting more than two years after the 2023 Saudi Commission for Health Specialties (SCFHS) regulatory update.
- According to this managerial cohort, organizational and structural barriers, including unclear job descriptions, staffing shortages, and resource deficits, are the primary constraints on full professional practice, while the training-to-practice gap and limited autonomy further hinder scope enactment.

**What are the implications of the main findings?**

- The findings highlight the need for institutional interventions, rather than policy changes alone, as the persistence of scope variability described by nurse managers and supervisors in this study suggests that regulatory publication alone may be insufficient for professional transformation in clinical settings.
- Operationalizing the 2023 SCFHS framework through revised job descriptions, simulation-based professional development, adequate staffing, and leadership-supported autonomy is essential to realize the intent of the national scope document and advance Saudi Vision 2030 healthcare objectives.

**Abstract:**

Background: In Saudi Arabia, nurses have long lacked a formally defined scope of practice, contributing to role ambiguity and role overlap. In 2023, the Saudi Commission for Health Specialties (SCFHS) released the first national Scope of Nursing and Midwifery Practice document. This study explored how nurse managers and supervisors perceived the scope of practice following this update in Makkah Cluster Hospitals. Methods: As the second, explanatory phase of a larger sequential explanatory mixed-methods study, a qualitative descriptive study was conducted between May and August 2025 with 20 nurses in managerial roles (Nursing Directors, Nursing Supervisors, and Head Nurses) at two hospitals within the Makkah Health Cluster. Participants were selected through purposive sampling, restricted to managerial and supervisory positions to capture a cross-departmental, organizational perspective on scope enactment. Interviews were conducted via Zoom in Arabic, translated into English, and analyzed using inductive thematic analysis consistent with the approach of Braun and Clarke. Trustworthiness was addressed through credibility, transferability, dependability, and confirmability, with reporting guided by the Consolidated Criteria for Reporting Qualitative Research (COREQ). Results: Four themes emerged from the accounts of the 20 participants (10 men and 10 women from both hospitals): (1) expanding the scope of nursing practice, (2) bridging the gap between training and practice, (3) overcoming barriers to full professional practice, and (4) promoting nurse empowerment and accountability. Participants described a tension between a formally defined scope and inconsistent enactment across departments and shifts. Unclear job descriptions, administrative constraints, staffing shortages, and resource deficits were the primary barriers, whereas autonomy and continuous development were identified as essential enablers. Conclusions: The nurse managers and supervisors in this study perceived the scope of nursing practice as variably enacted and frequently constrained following the 2023 SCFHS update. These findings suggest that regulatory publication alone may be insufficient, highlighting the need to operationalize the framework through revised job descriptions, simulation-based professional development, adequate staffing, and leadership-supported autonomy aligned with Saudi Vision 2030. Because these findings reflect the perspectives of nursing leaders rather than bedside clinical nurses, their application to direct patient care contexts should be interpreted with appropriate caution.

## 1. Introduction

### 1.1. Background and Significance

The scope of nursing practice encompasses the legal, ethical, and professional boundaries within which nurses operate, influencing their ability to provide high-quality care to patients [1]. A well-defined and supportive scope of practice helps nurses make the most of their abilities, enhancing job satisfaction, improving patient outcomes, and increasing retention rates [2]. In recent years, growing healthcare challenges, such as workforce shortages and the rising complexity of patient care, have made efforts to broaden and clarify the scope of nursing practice increasingly important [3].

The Queensland Nursing Council broadly defines the scope of nursing practice as the activities that nurses are educated, authorized, and qualified to undertake [4]. Similarly, the Nursing and Midwifery Board of Ireland defines it as the range of roles, functions, responsibilities, and activities that a registered nurse is educated, competent, and authorized to perform [5]. Globally, the scope of nursing practice has been shaped by advancements in healthcare, educational reforms, legal frameworks, and growing demands for quality patient care, evolving from basic caregiving to include complex clinical decision-making, patient education, and leadership functions [6].

A clearly defined scope of practice increases nurses’ professional autonomy and confidence, promotes greater job satisfaction, and enhances their ability to apply their knowledge and skills, make independent clinical judgments, and contribute meaningfully to patient care [7]. Conversely, when the scope of practice is restrictive, poorly defined, or hierarchically constrained, nurses may experience role ambiguity, reduced professional fulfillment, and compromised patient outcomes [8]. Understanding these dynamics is essential for healthcare organizations seeking to build resilient, high-performing nursing workforces.

These dynamics align with role theory, which conceptualizes role ambiguity as a condition that arises when an individual lacks clear, consistent information about the expectations, boundaries, and authority attached to an organizational position [9]. Applied to nursing, role ambiguity emerges when the formally defined scope of practice diverges from the scope of practice actually enacted in daily clinical work, leaving nurses uncertain about which activities they are authorized, competent, and expected to perform. This study draws on role theory, together with the conceptual model underlying the Actual Scope of Nursing Practice (ASCOP) framework, which operationalizes scope of practice across domains such as assessment, care planning, supervision, and professional autonomy [10], as an interpretive lens for understanding how nurse managers and supervisors described the gap between the formally defined 2023 SCFHS scope and its enactment in clinical practice.

### 1.2. The Scope of Nursing Practice: International Context

Internationally, the scope of nursing practice has expanded considerably in response to evolving healthcare system demands, technological advancements, and increasing patient complexity. In many countries, nurses now hold advanced degrees, prescribe medications, and lead public health initiatives [11]. However, in some healthcare systems, this progress is hindered by structural challenges and institutional resistance, limiting nurses’ ability to practice to the full extent of their training and expertise [12].

Advanced practice nurse (APN) subgroups under the International Council of Nurses have conducted periodic global assessments showing that the implementation of APNs may facilitate universal health coverage and the achievement of the Sustainable Development Goals, yet inconsistent regulation, education standards, and scope definitions across nations remain significant barriers [13]. In many jurisdictions, physician oversight is required for nurse practitioners to practice and prescribe, restricting their capacity to provide care independently [14].

In the Middle East and North Africa (MENA) region, several countries have initiated healthcare reforms to improve access to quality medical treatment, yet significant obstacles remain. Cultural influences, including the prioritization of family involvement over individual patient engagement and physician-dominated care models, continue to shape how healthcare professionals interact and what roles nurses are permitted to assume [15]. Developing clear definitions, educational criteria, legislation, and a distinct scope of practice is recognized as an essential first step toward establishing advanced practice nursing roles in Arab nations of the Eastern Mediterranean Region [16].

### 1.3. The Scope of Nursing Practice in Saudi Arabia

Saudi Arabia presents a particularly instructive case for studying the scope of nursing practice. As the country undergoes a substantial healthcare transformation under Saudi Vision 2030, the nursing profession has been called upon to expand its roles and responsibilities. For many years, however, a formally defined and differentiated scope of practice for different nursing categories was absent. The SCFHS registered nurses, based on educational level alone, designated diploma-level graduates as nursing technicians and baccalaureate graduates as nursing specialists, without providing corresponding scope-of-practice definitions aligned with these designations [17,18]. This created a regulatory vacuum in which role overlap across categories was structurally embedded, and role ambiguity was pervasive.

Aljohani et al. (2022) conducted a landmark descriptive cross-sectional study using the Arabic Actual Scope of Nursing Practice (A-ASCOP) questionnaire [10,18] among 928 nurses across Saudi Arabia. They found an overall A-ASCOP mean of 4.59 out of 6, with significant variation by nationality, gender, employment program, and educational level. Crucially, the absence of clearly defined internal scope-of-practice regulations did not appear to influence nursing duties, as nurses across categories performed similar activities, highlighting the urgent need for government-mandated scope definitions. The study concluded that clearly defining the scope of practice for nurses is essential for a country undergoing major healthcare transformation [18].

Hamadi, Aboshaiqah, and Alanazi (2024) further confirmed these findings in Ministry of Health hospitals in Al-Hasa province, where data collected in 2021 from 286 nurses revealed no statistically significant differences in overall ASCOP mean scores across health assistants, nursing technicians, nursing specialists, and senior nursing specialists (overall mean = 4.64/6) [4]. This lack of differentiation was interpreted as evidence of the absence of distinct scopes of practice for the different nursing categories, resulting in role overlap. The study recommended that policymakers develop clearly defined, category-specific scopes of practice and reconsider the utility of maintaining multiple nursing categories if they perform essentially the same functions [4].

A critical regulatory milestone was reached in 2023 when the SCFHS Nursing and Midwifery Professional Council (NMPC) published the first national Scope of Nursing and Midwifery Practice document [19]. The document was the product of a multi-year process (2019–2022) involving a systematic literature review, benchmarking against international frameworks, and a national stakeholder workshop with nurses, midwives, educators, and hospital leaders. The resulting framework defined the scope of practice for four categories—Nurse Technician, Nurse Specialist, Midwife Technician, and Midwife Specialist—across eight practice domains, explicitly linking each category to its corresponding educational level and competency. Aldossary (2013) noted that Saudi nurses continue to lack a clearly legitimized role identity in practice, with supervisory authority or inter-group agreements partially filling the regulatory gap [20]. The most recent study conducted in Makkah on nursing professional factors (Rawah and Banakhar, 2022) highlighted the need for future work examining the scope of practice, work environment, and the influence of policy and leadership on professional nursing roles within the Makkah Health Cluster [21].

### 1.4. Rationale and Study Aim

Despite the landmark publication of the 2023 SCFHS Scope of Nursing and Midwifery Practice document, the ways in which this regulatory development has been experienced, interpreted, and enacted by nurses in clinical and managerial leadership positions have not been qualitatively investigated. The existing post-2023 literature on the scope of nursing practice in Saudi Arabia focuses on advanced practice nursing roles [22,23], policy analysis [24], and nurse practitioner role perspectives [25], leaving the lived experiences of nursing leaders responsible for overseeing scope enactment in hospital settings largely unexplored. Research specifically examining these perceptions within the Makkah Health Cluster, a unique cluster-hospital administrative model, is similarly absent.

Although quantitative studies have documented scope enactment scores and role overlap in the pre-2023 period, the meaning nurses attach to their practice boundaries after the SCFHS regulatory update, the specific barriers they encounter in operationalizing the new framework, and their aspirations for professional autonomy have not been systematically explored through in-depth qualitative research. The present study addresses this gap directly by analyzing data collected between May and August 2025, over two years after the SCFHS document’s release. To the best of our knowledge, it is among the first qualitative investigations of nurse managers’ and supervisors’ perceptions of the scope of practice following this regulatory update, with a specific focus on enabling and constraining factors affecting its clinical enactment. The study is guided by the following research question:

*In the context of the 2023 SCFHS Scope of Nursing and Midwifery Practice framework, what are the perceptions and experiences of nurse managers and supervisors regarding the scope of practice in Makkah Cluster Hospitals, and what factors enable or constrain its full enactment among the nursing staff under their oversight?*

## 2. Methods

### 2.1. Study Design

A qualitative descriptive design was employed to explore how nurse managers and supervisors perceive and experience the scope of nursing practice, with reporting guided by the Consolidated Criteria for Reporting Qualitative Research (COREQ) [26], a 32-item checklist developed for studies based on individual interviews and focus groups. The qualitative component reported in this manuscript formed the second phase of a larger sequential explanatory mixed-methods study. The first, quantitative phase comprised a cross-sectional online survey of 259 nurses at the same Makkah Cluster hospitals, using the A-ASCOP, the Organizational Commitment Questionnaire, and the Practice Environment Scale of the Nursing Work Index. That phase yielded a high A-ASCOP mean score, indicating that nurses broadly self-reported performing a wide range of nursing activities, with the lowest subscale mean observed for the Integration and Supervision of Staff subscale (M = 5.39, SD = 0.84). However, a high overall level of self-reported scope enactment does not explain the organizational mechanisms behind it, nor the variability and constraints that may coexist with high activity scores.

The qualitative phase was designed to provide in-depth insights that elaborated on and contextualized these quantitative findings from the first phase, with particular attention to the organizational and managerial perspective on scope enactment, given the lower score in the first phase for supervisory and integrative functions. Data were collected between May and August 2025, more than two years after the publication of the 2023 SCFHS Scope of Nursing and Midwifery Practice document. This timing positioned the study within the post-regulatory context, enabling it to capture how nurse managers and supervisors perceive the scope of practice as it is enacted under the new national framework. The qualitative methodology aligns with the study’s aim to capture the richness and complexity of participants’ subjective professional experiences in a culturally and structurally specific context.

### 2.2. Setting

The study was conducted at two hospitals within the Makkah Health Cluster in Makkah City, Saudi Arabia: Hera General Hospital and King Faisal Hospital. These institutions operate under a unified cluster hospital administrative model, a healthcare configuration introduced to enhance operational efficiency and integration across facilities. Makkah City presents a distinctive healthcare context because of its status as a pilgrimage destination requiring surge-capacity healthcare provision, alongside year-round demands on the regional healthcare system.

### 2.3. Participants and Sampling

A purposive sampling strategy was used to recruit participants who could provide informed, experience-rich insights into the scope of nursing practice from positions of professional authority. This sampling strategy intentionally focused on nurses in managerial and supervisory positions rather than bedside clinical nurses. Nurse managers and supervisors oversee clinical practice across multiple departments, units, and shifts, giving them a broader organizational perspective on how the scope of nursing practice is implemented. By contrast, bedside nurses typically experience scope of practice within their own clinical unit and daily responsibilities.

The quantitative phase of the larger study had already captured frontline nurses’ self-reported scope enactment from a separate sample of 259 nurses. Therefore, the qualitative phase was designed to complement these findings by exploring organizational and managerial factors, such as role clarity, staffing allocation, and delegation of authority, that nurse managers are well-positioned to describe. Because managers and supervisors also have clinical experience while overseeing nursing practice, they can provide insights into both direct patient care and the organizational factors that influence scope enactment. However, this sampling approach does not include the perspectives of bedside nurses, whose day-to-day experiences may offer additional insight into the practical challenges of implementing the scope of nursing practice.

Twenty nurses serving in managerial and supervisory roles were selected, including nursing directors, nursing supervisors, and head nurses. All participants were currently employed at either Hera General Hospital or King Faisal Hospital. Nurses who had participated in the quantitative phase of the study were excluded, ensuring that the qualitative participants represented an independent perspective. Inclusion criteria required participants to be registered nurses holding managerial or supervisory roles so that they could comment on nursing practice scope from both personal clinical experience and an organizational oversight perspective. Recruitment was facilitated by nurse managers working at the study hospitals. Sample size was determined by data saturation; interviews continued until no new themes or conceptual content emerged from the data.

### 2.4. Data Collection

Semi-structured interviews were conducted online via Zoom between May and August 2025. Each interview lasted between 30 and 60 min. Interviews were conducted in Arabic to allow participants to express their views clearly and comfortably in their first language. The discussions were subsequently translated into English by a bilingual expert with familiarity with the Saudi nursing context. A forward translation was performed for each transcript. The interview guide was developed by the principal investigator based on the study’s aims and relevant literature. It was also reviewed by the research supervisor for content coverage and clarity prior to use. The full semi-structured interview guide is provided in the Supplementary Materials (File S1). The guide was not piloted with a separate group of nurses before data collection. This methodological decision is acknowledged as a limitation. The interview guide was used to elicit participants’ perspectives on the scope of nursing practice in their institutions, including how it is defined, applied, and experienced in daily clinical work; what factors enable full professional practice; and what constraints or barriers are encountered. Follow-up probing questions, such as “Why do you think this is the case?” and “Can you elaborate on that?”, were posed to elicit greater depth and contextual detail. Participants were informed that their responses would remain confidential.

### 2.5. Data Analysis

Interviews were audio-recorded and transcribed verbatim. Data were analyzed using inductive thematic analysis following the six-phase process described by Braun and Clarke [27], without applying a predefined coding framework. The principal investigator listened to each audio recording and read the transcripts several times to become familiar with the data and ensure an accurate understanding of participants’ accounts. All transcripts were organized in a Microsoft Word document for coding and comparison across participants. Dedicated qualitative analysis software, such as NVivo, was not used, as the dataset of 20 interviews was manageable for manual analysis. Initial codes were generated inductively through line-by-line coding and reviewed by the principal investigator. As coding was undertaken by a single researcher, discussions with the research supervisor were used to strengthen the credibility and dependability of the analysis. The supervisor independently reviewed selected transcript excerpts, examined the consistency between the data, codes, and emerging themes, and provided feedback throughout the analytic process. Related codes were then grouped into broader categories, and their relationships were examined to identify overarching themes. This process resulted in four main themes, presented in the Section 3. No additional subthemes emerged beyond the descriptive categories presented in the Results (Section 3.2). The final themes were reviewed, refined, and agreed upon through ongoing discussions between the principal investigator and the research supervisor to ensure they accurately reflected the participants’ experiences.

### 2.6. Trustworthiness and Rigor

The study adhered to established qualitative research standards for ensuring credibility, transferability, dependability, and confirmability [28]. Credibility was strengthened through prolonged engagement with the data (multiple verbatim readings of each transcript by the principal investigator), member checking with participants (a summary of the preliminary themes was shared with participants by email to confirm that the interpretation reflected their intended meaning), and peer debriefing with the research supervisor (iterative review of codes, themes, and supporting excerpts). Transferability was supported by providing rich descriptions of the study context and participant characteristics. Dependability was addressed through the maintenance of an audit trail, consisting of dated records of coding decisions, definitions of codes and themes, and analytic memos produced as themes were refined, which together documented methodological decisions. Confirmability was ensured by grounding interpretations in direct participant quotations and maintaining reflexive awareness throughout the analytic process.

### 2.7. Reflexivity

The principal investigator (H.M.A.) was a nurse employed within the Makkah Health Cluster and therefore occupied an insider position relative to the study setting. This positionality offered contextual familiarity that facilitated rapport and interpretation of institution-specific terminology, though it also carried the risk that prior assumptions about organizational dynamics could shape data collection or interpretation. The principal investigator addressed this by maintaining reflexive notes throughout data collection and analysis to document and bracket pre-existing assumptions. The research supervisor (A.K.), who was not embedded within the Makkah Health Cluster administrative structure, served as an external auditor of the coding and theme development process, reviewing transcript excerpts independently of the principal investigator’s prior institutional knowledge. Participants were informed that although the principal investigator was professionally affiliated with the Makkah Health Cluster, the investigator did not have a direct supervisory role over either of the two study hospitals or the participants themselves.

### 2.8. Ethical Considerations

The study was conducted in accordance with the World Medical Association’s Declaration of Helsinki and approved by the Institutional Review Board of King Saud University (Ref No.: KSU-HE-25-694; approval dated 13 May 2025). Additional approval was obtained from the participating hospitals as required. Participation was entirely voluntary, and informed consent was obtained from all participants prior to data collection. Participants were fully informed of the study’s purpose, the procedures involved, and their right to withdraw at any time without consequence. Confidentiality was maintained by assigning unique participant codes, and all data were stored securely with access restricted to the research team.

## 3. Results

### 3.1. Participant Characteristics

A total of 20 nurse managers and supervisors participated in the qualitative phase, with an equal distribution of men (n = 10, 50%) and women (n = 10, 50%). Participants’ ages ranged from 33 to 45 years, with a mean age of 38.65 years (SD = 3.21), indicating that the majority were mid-career professionals with substantial nursing management experience. Most participants held a master’s degree (n = 14, 70%), while the remaining participants held a bachelor’s degree (n = 4, 20%) or a PhD (n = 2, 10%), reflecting a highly educated managerial cohort.

Participants were drawn from both study hospitals: 45% (n = 9) from Hera General Hospital and 55% (n = 11) from King Faisal Hospital, ensuring representation from different institutional contexts within the Makkah Health Cluster. All participants served in managerial roles (nursing directors, nursing supervisors, and head nurses) that positioned them to provide informed perspectives on both individual nursing practice and the organizational factors shaping the scope of practice. Table 1 summarizes the participants’ demographic characteristics.

### 3.2. Overview of Themes

Following transcription and thematic analysis of the recorded interviews, four dominant themes directly pertaining to the scope of nursing practice were identified. These themes captured key dimensions of participants’ experiences regarding how the scope of practice is conceptualized, enacted, constrained, and framed within their institutions. Table 2 provides an overview of the themes. No additional formal subthemes emerged beyond the descriptive content presented under each main theme.

### 3.3. Theme 1: Expanding the Scope of Nursing Practice

Participants noted that although the scope of nursing practice is generally well defined at a conceptual and policy level, its practical application varies across departments, shifts, and levels of authority. Several nurses reported that when they were empowered to fully utilize their skills, the quality of patient care improved substantially. Others emphasized the need for a standardized scope of practice that aligns with service levels and evidence-based role expectations to minimize role ambiguity. Participants also stressed the importance of regularly updating scope-of-practice guidelines to reflect evolving competencies and professional standards. Overall, clarity, empowerment, and professional autonomy were identified as essential components of effective nursing practice.

Participants expressed that empowerment to practice to their full scope was not uniformly experienced across the institution. Nurses explained that the extent to which they could exercise their competencies depended on the department in which they worked, the shift to which they were assigned, and the attitudes of those in authority over them. This inconsistency was a source of frustration:

“When we’re allowed to use our skills fully, patient care improves significantly.” (P8)

“The scope should fit the service level and evidence-based roles rather than depend on individual interpretation.” (P5)

These accounts reflect a broader tension between the formal articulation of the scope of nursing practice and its actual enactment in clinical practice. Participants expressed a strong desire for institutional leadership to develop unified, evidence-informed scope-of-practice guidelines to be applied consistently across all departments and shifts, reducing the discretionary nature of role boundaries.

### 3.4. Theme 2: Bridging the Gap Between Training and Practice

Several participants emphasized that while their theoretical education provided a solid foundation, significant challenges were encountered in translating this knowledge into clinical practice. The gap between what nurses were taught and what they were expected or permitted to do in practice was described as a persistent impediment to full scope enactment. Nurses highlighted the importance of ongoing professional development, particularly hands-on training through simulations, practical workshops, and guided clinical sessions, rather than relying on theoretical assessments or written checklists. Continuous learning and periodic competency reassessment were viewed as essential for maintaining confidence, enhancing clinical judgment, and ensuring the consistent delivery of safe and effective patient care. Participants expressed concern that existing evaluation methods were insufficient to assess real-world clinical competency:

“We always need more educational programs and continuous learning.” (P1)

“The last evaluation methods relied mainly on written checklists instead of practical demonstration.” (P5)

“Nurses generally receive sufficient general and specialized competencies.” (P13)

These accounts collectively highlight that the development of the scope of nursing practice is not only a matter of policy definition but also depends on educational infrastructure. Even when the scope of practice is formally defined, nurses must be continuously supported through practical training to develop the confidence and competence to enact it. Participants indicated that simulation-based and clinically supervised learning approaches were particularly valued as mechanisms for translating scope-of-practice expectations into consistent clinical performance.

### 3.5. Theme 3: Overcoming Barriers to Full Professional Practice

Participants consistently identified a cluster of organizational and systemic barriers that hindered their ability to practice to the full extent of their professional competencies. Unclear job descriptions, administrative constraints, and limited resources were the most frequently cited impediments. Staffing shortages and inadequate equipment were described as factors that directly affected the quality of care and contributed to elevated stress levels among nursing staff.

Role ambiguity, arising from imprecise job descriptions and insufficient delegation of decision-making authority, was experienced as a fundamental constraint. Participants described being aware of their clinical capabilities but unable to apply them because of institutional structures that had not kept pace with the profession’s development:

“Unclear job descriptions and lack of authority in decision-making can limit our practice.” (P3)

“Administrative restrictions limit our ability to apply our full professional scope.” (P4)

“Lack of equipment and unequal task distribution make it difficult to perform effectively.” (P12)

These barriers were not understood by participants as inevitable features of the clinical environment, but rather as organizational deficiencies that could be addressed. Participants emphasized that addressing administrative barriers and ensuring adequate resource allocation would significantly enhance the quality of care and staff satisfaction. The accounts reflected a workforce that perceived itself as capable of making a greater professional contribution but was systemically constrained from realizing it.

### 3.6. Theme 4: Promoting Nurse Empowerment and Accountability

Participants articulated a vision of an effective scope of nursing practice characterized by three intersecting elements: autonomy, accountability, and evidence-based care. They described the ability to practice independently within their competencies while maintaining meaningful collaboration with other healthcare disciplines as fundamental to both patient outcomes and professional satisfaction. An important nuance in participants’ accounts was the understanding that autonomy and accountability were not opposing forces but complementary principles; nurses sought the freedom to exercise professional judgment within clearly defined and mutually understood boundaries.

The capacity for professional growth was also prominently featured, with participants emphasizing that the scope of practice must be dynamic enough to accommodate career development without being so flexible as to eliminate the structural clarity that protects both nurses and patients:

“A good scope should include autonomy, accountability, and evidence-based care.” (P3)

“Nurses should practice independently within their competence and collaborate with other disciplines.” (P10)

“The scope must be flexible enough to allow professional growth but structured enough to prevent overstepping boundaries.” (P7)

Participants also noted that empowering nurses to practice within an appropriately defined scope fostered a sense of professional identity and organizational belonging. This dimension of the findings highlights the bidirectional relationship between scope clarity and workforce well-being: nurses who experienced clarity and empowerment in their scope of practice described greater professional fulfillment, as well as increased motivation, to contribute to the quality of care and patient safety.

Participants’ accounts were highly consistent across all four themes, with no major differences or contradictory views regarding the implementation of the scope of nursing practice or the factors influencing it. This consistency appears to reflect the characteristics of the study sample rather than limitations in the analysis. All 20 participants held managerial or supervisory positions within the same two-hospital cluster; therefore, they shared similar organizational responsibilities and exposure to the same policies, staffing practices, and administrative processes. As a result, their experiences and perceptions were largely aligned. Data collection continued until thematic saturation was reached, meaning that no new concepts or themes emerged in additional interviews. Consequently, the findings primarily reflect the shared experiences of nurse managers and supervisors rather than individual or isolated perspectives.

## 4. Discussion

This qualitative study explored how nurse managers and supervisors perceive and experience the scope of nursing practice in Makkah Cluster Hospitals. Thematic analysis of semi-structured interviews with 20 nurse managers and supervisors yielded four interconnected themes: expanding the scope of nursing practice; bridging the gap between training and practice; overcoming barriers to full professional practice; and promoting nurse empowerment and accountability. Taken together, these themes indicate that this cohort of managers was intellectually and professionally aware of what an effective scope of practice entails, yet routinely described the nursing staff under their oversight as constrained by organizational, administrative, and resource-related barriers to practicing to their full scope.

This study makes several important contributions to the literature. First, it is among the first qualitative studies conducted following the 2023 update to the SCFHS Scope of Nursing and Midwifery Practice, providing early evidence on how the updated framework is being implemented within healthcare organizations in Saudi Arabia. Unlike earlier Saudi studies that primarily described the lack of clear role boundaries before the updated framework, this study explores how the revised scope of practice is being translated into everyday clinical and organizational practice [4,18,20]. Second, the study focuses on nurse managers and supervisors, offering an organizational perspective that has received limited attention in previous ASCOP research, which has largely examined the experiences of frontline nurses [4,18]. Managers are well-positioned to identify factors that support or hinder the implementation of the updated scope of practice. Finally, whereas previous studies primarily described role overlap, this study identifies the organizational factors that influence successful scope enactment. These include role clarity, delegation of authority, staffing allocation, and performance evaluation processes. By examining how these organizational factors shape the implementation of the national framework, the study provides a deeper understanding of why translating policy into routine clinical practice remains challenging in some settings.

Participants’ accounts of variability in scope enactment across departments, shifts, and authority levels resonate strongly with existing literature and are particularly significant given the post-2023 regulatory context in which this study was conducted. The 2023 SCFHS Scope of Nursing and Midwifery Practice document established a national framework explicitly intended to standardize practice across nursing categories; yet the findings from this study, collected more than two years after its publication, suggest that its intent has not yet been uniformly translated into daily clinical practice, at least as perceived by the nurse managers and supervisors interviewed. Similarly, a Spanish study found that psychological demand, practice environment, and individual development need strength were all positively correlated with a broader enacted scope, while unresolved role ambiguity was identified as a limiting factor [29]. The present findings extend this insight to the post-regulatory Saudi context, suggesting that even where a formal framework exists, nurses’ practical experience of scope boundaries, as described by their managers and supervisors, remains fragmented and contingent on local leadership attitudes rather than on unified institutional operationalization of the national framework.

Participants’ call for a standardized, evidence-based scope of practice aligned with service-level expectations echoes the recommendations of Aljohani et al. (2022) and Hamadi, Aboshaiqah, and Alanazi (2024), who documented role overlap and the absence of meaningful scope differentiation across nursing categories in Saudi Ministry of Health hospitals [4,18]. Unlike these earlier studies, participants in the present study also articulated possible solutions: clarity, standardization, and evidence-based role definitions. This is consistent with the broader international evidence that a well-defined scope enhances role clarity, professional identity, and patient care quality [1,7].

Beyond variability in scope enactment, the findings also revealed a persistent gap between theoretical education and clinical practice as described by participants. The theme of bridging the gap between training and practice reflects a widely documented challenge in nursing workforce development. Participants explained that theoretical competency does not automatically translate into clinical confidence, particularly in systems in which evaluation methods have relied on written assessments rather than the demonstration of practical skills. This finding aligns with international evidence that practical, simulation-based, and supervised clinical training is essential for enabling nurses to enact their scope of practice confidently [30]. The emphasis on continuous professional development is also consistent with a study by Gottlieb, Gottlieb, and Bitzas (2021), who demonstrated that empowering nurses through education and institutional change is essential for strengthening healthcare systems [31].

In the Saudi context, this gap is compounded by the historical absence of a formal scope-of-practice framework, which may have created uncertainty about which clinical behaviors nurses are not only permitted to perform but also expected to demonstrate. Without a clear regulatory anchor, the training-to-practice gap becomes both a clinical and an organizational challenge: nurses may be educationally prepared for practices that institutional culture has not yet legitimized.

Compounding both the variability and the training-to-practice gap, participants consistently identified a cluster of organizational and systemic barriers, including unclear job descriptions, administrative restrictions, staffing shortages, and resource deficits, which represent a complex interplay of systemic and structural factors. These findings are consistent with those of van Kraaij et al. (2024), who documented that organizational rigidity and competing demands create conditions in which nurses are unable to practice to their full potential, even when they possess the requisite competencies [8]. Similarly, Oshodi and Sookhoo (2024) identified inadequate staffing as a structural constraint on nursing competency development and full scope enactment [32]. Role ambiguity as a barrier to practice is well established in the literature. Sariköse and Göktepe (2022) found that nurses’ individual, professional, and work environment characteristics all contributed to job performance, with unclear roles limiting effective practice [33]. In the Saudi context, the absence of formalized scope-of-practice definitions has historically created structural role ambiguity at a systemic level [18], which may amplify the effects of unit-level administrative inconsistencies described by participants in the present study. Aldossary (2013) reinforced this, noting that nurses in Saudi Arabia must challenge conventional wisdom about their place in acute care to make a substantial impact on population health [20].

Participants’ emphasis on the correctable nature of these barriers is notable. They did not frame resource deficits or administrative constraints as immutable; rather, they identified specific organizational investments, clearer job descriptions, resource allocation, and equitable task distribution as measures that would meaningfully improve their capacity to practice to their full scope. This constructive orientation suggests that a motivated nursing workforce awaits the institutional conditions necessary to fulfill its professional potential.

The findings raise an important question: Why do these barriers remain more than two years after the publication of the national Scope of Nursing and Midwifery Practice framework? The present findings, together with existing evidence, suggest several possible explanations. First, there appears to be a gap between the national framework and its implementation at the organizational level. Although the SCFHS document clearly defines the scope of practice for different nursing categories, hospitals must translate these standards into clear job descriptions, delegation processes, and performance evaluation systems. Participants consistently identified unclear job descriptions as an ongoing challenge, suggesting that the implementation process remains incomplete.

Second, scope-of-practice enactment appears to vary across clinical settings. Participants reported that role boundaries often depended on the interpretation of local managers and organizational practices rather than on consistent organizational implementation. This finding suggests that without active organizational oversight and monitoring, the implementation of the national framework may rely largely on local leadership and workplace culture.

Finally, organizational factors such as staffing shortages and limited resources continue to influence nurses’ ability to practice to their full scope. Even when roles are clearly defined, nurses may be unable to perform all aspects of their scope of practice if adequate staffing, equipment, and organizational support are not available. These findings indicate that the successful implementation of the national framework depends on regulatory guidance as well as organizational readiness and resource availability. Together, these findings demonstrate that scope enactment is influenced by factors at multiple levels. National policies provide the regulatory foundation, but hospital leadership, organizational processes, and available resources determine how these policies are translated into everyday clinical practice.

Despite these constraints, participants identified a clear and aspirational professional vision. The fourth theme’s articulation of the relationship between autonomy, accountability, and evidence-based practice reflects a sophisticated professional ethos among the nursing managers who participated in this study. Their vision of the scope of practice as simultaneously structured and flexible, providing the freedom for professional growth while preventing the overstepping of boundaries, corresponds to the international literature on advanced practice nursing and professional autonomy. Lockwood and Schober (2024) identified several factors influencing nurse practitioners’ clinical autonomy from a self-determining perspective, emphasizing that autonomy is a product of regulatory permission as well as professional identity, institutional culture, and collaborative relationships with other disciplines [34].

The observation that nurses who exercise autonomy within their scope experience greater professional fulfillment is consistent with Faridi et al. (2025), who found a positive association between professional autonomy and clinical happiness among nurses [35]. Gottlieb, Gottlieb, and Bitzas (2021) described a strengths-based model of nursing and healthcare leadership in which workplace autonomy and agency are viewed as foundational to professional flourishing [31]. The present findings ground these theoretical arguments in the specific institutional and cultural context of Makkah Cluster Hospitals, demonstrating that Saudi nurse managers aspire to and value such conditions.

The findings should also be considered within the broader social and organizational context of the Saudi healthcare system. Although these factors were not explored directly in this study, previous research suggests that they may influence how the scope of nursing practice is implemented. Historically, healthcare in Saudi Arabia has been organized around a physician-led model, in which nurses have had less clearly defined professional authority. Before the publication of the 2023 SCFHS Scope of Nursing and Midwifery Practice framework, nurses’ roles were often shaped by local organizational practices and supervisory decisions rather than by a nationally standardized scope of practice [20]. While the updated SCFHS framework was introduced to address this issue, the present findings suggest that differences in implementation may still exist across healthcare organizations. Similarly, Abbas et al. (2022) noted that organizational structures within physician-led healthcare systems may continue to limit nurses’ ability to practice independently despite regulatory changes [12].

Workforce characteristics may also influence scope enactment. Previous Saudi studies have reported differences in ASCOP scores according to gender and nationality, suggesting that these factors may affect how nurses understand and enact their professional roles [4,18]. Given that the Saudi nursing workforce includes both Saudi and expatriate nurses, differences in educational background, professional experience, and organizational expectations may contribute to variation in scope enactment. However, in the present study, these issues did not emerge as major themes. The sample included an equal number of male and female participants, and recruitment was not based on nationality. Instead, participants emphasized organizational and administrative factors as the primary influences on scope enactment. This finding may indicate that managerial responsibilities render organizational barriers more visible than workforce characteristics. Because the interview guide did not specifically explore gender or nationality, future qualitative studies should examine how these factors interact with organizational structures and influence the implementation of the scope of nursing practice in Saudi Arabia.

### 4.1. Study Implications

These findings carry important implications at multiple levels of the healthcare system. The implications presented below are based on the study findings and the authors’ interpretation of participants’ experiences within their organizational context. Although the participants’ accounts provide valuable insights into the implementation of the scope of nursing practice, the recommended actions should be viewed as informed suggestions rather than conclusions that can be established based on the qualitative data.

At the level of nursing practice, healthcare organizations should develop clear, standardized, and evidence-based scope-of-practice guidelines that apply uniformly across all departments and shifts, reducing the discretionary variation in current role enactment identified by participants. Such guidelines should be grounded in the professional competencies expected of each nursing category and periodically revised to reflect evolving clinical standards. The transition from informal practice boundaries to formally enacted scope requires robust institutional support. Nurses must be empowered to practice within their defined scope through organizational policies that clarify decision-making authority, ensure the equitable distribution of responsibilities, and eliminate unnecessary administrative barriers. Interprofessional collaboration should be explicitly incorporated into scope-of-practice frameworks. Participants identified independent practice within their competencies, alongside collaboration with other healthcare disciplines, as the ideal model, suggesting that an effective scope of practice is not an isolated professional claim but a negotiated relational practice.

At the managerial and policy level, the present findings deliver an equally pointed message to hospital administrators and nursing leaders: the existence of the 2023 SCFHS Scope of Nursing and Midwifery Practice document does not automatically produce change at the bedside, at least as perceived by the nurse managers and supervisors in this study. The document itself explicitly states that the scope of practice shall be used in practice settings as a foundation for nursing roles, professional development, and performance appraisal [19], yet the accounts of the nurse managers and supervisors, collected two years after its release, describe experiences of persistent role ambiguity, administrative constraint, and variable enactment that suggest this foundational work may not have been completed. Therefore, hospital administrators might consider translating the SCFHS framework into institutional practice, recognizing that this proposal extends beyond what the qualitative evidence alone can establish as causally necessary, by revising job descriptions to reflect the four-category scope distinctions, updating delegation and decision-making authority frameworks to align with Nurse Technician and Nurse Specialist competency boundaries, and embedding scope-of-practice criteria into performance appraisal systems.

The consistent identification of staffing shortages and resource deficits as barriers to the full scope of practice also has direct policy implications. Adequate staffing ratios, fair workload distribution, and access to the necessary clinical equipment are not merely operational concerns but preconditions for realizing the professional scope that regulatory bodies have sought to define. Healthcare policy, in alignment with Saudi Vision 2030’s goals of empowering healthcare professionals, must address these structural enablers alongside regulatory definitions [18].

The cluster hospital model itself warrants particular attention. The administrative unification of hospitals within a health cluster offers an opportunity to achieve consistency in scope-of-practice enactment across facilities, but this opportunity will only be realized if cluster-level leadership actively champions standardized role definitions and provides the resources necessary for their enactment.

Finally, the training-to-practice gap identified in this study has direct implications for nursing education programs, though this recommendation follows from participants’ reported experiences rather than from a tested intervention. Educators should prioritize practical, simulation-based, and clinically supervised learning over exclusively theoretical approaches to competency assessment. Regular skill assessments and continuous professional development activities that require nurses to demonstrate competency in real or simulated clinical settings are essential for supporting full scope enactment. Nursing schools should also play a leading role in preparing graduates to practice within the scope of practice [18].

### 4.2. Strengths and Limitations

This study offers several methodological strengths. The use of purposive sampling to recruit experienced nurse managers and supervisors ensured that participants could provide professionally grounded, contextually rich insights into scope-of-practice dynamics from both personal clinical experience and organizational oversight perspectives. The use of Arabic as the interview language eliminated language-related barriers and allowed participants to express their views with precision and nuance. Equal representation of male and female participants and the inclusion of participants from two hospitals within the Makkah Health Cluster enhance the breadth and representativeness of the findings within the study context.

However, several limitations must be acknowledged when interpreting the results. The qualitative phase included a relatively small sample of 20 participants from two hospitals within a single health cluster in Makkah City. Although data saturation was achieved and sample size was determined according to established qualitative principles, the findings may not be fully transferable to other hospitals, health clusters, or regions within Saudi Arabia, where cultural, organizational, and administrative characteristics may differ. Only nurses in managerial and supervisory positions were included. This sampling approach provided valuable organizational insights; however, it did not capture the perspectives of bedside clinical nurses who implement the scope of nursing practice in their daily patient care. Nurse managers are more likely to identify organizational, administrative, and policy-related challenges, whereas bedside nurses may experience additional barriers related to direct patient care, interprofessional collaboration, and everyday clinical practice. Therefore, the findings should be interpreted as reflecting the perspectives of nurse managers and supervisors rather than those of frontline nurses, who may hold distinct perceptions of scope enactment.

Although conducting interviews via Zoom supported logistical convenience and safety, it may have limited the development of interpersonal rapport and restricted the communication of nonverbal cues that sometimes enrich qualitative findings. Furthermore, interviews were conducted in Arabic and translated into English through a single forward translation by a bilingual expert familiar with Saudi nursing terminology. No independent back-translation or formal translation validation procedure was performed, which introduces a potential layer of translation between participants’ original expressions and the reported quotations, despite the translator’s familiarity with the Saudi nursing context.

Data coding and theme development were conducted by a single principal investigator. To strengthen the credibility and dependability of the analysis, the coding process and emerging themes were reviewed and discussed regularly with the research supervisor. However, because only one researcher performed the coding, formal inter-coder reliability could not be assessed. In addition, the interview guide was reviewed by the research supervisor for content and clarity. However, it was not piloted with a separate group of nurses before data collection, which may have limited the opportunity to refine the wording and sequencing of the questions in advance. The consistency observed across participants’ responses may also be related to the similar managerial and supervisory roles held by all participants. Including nurses from a wider range of clinical and organizational positions could have provided a broader diversity of experiences and perspectives regarding scope enactment. Finally, the study was conducted at a specific historical moment when the formal scope-of-practice framework for Saudi nursing was newly established. Longitudinal research examining how nurses’ perceptions and experiences of scope enactment change as the regulatory and institutional framework matures would significantly advance understanding of this dynamic.

## 5. Conclusions

This qualitative study provides evidence that nurse managers and supervisors in Makkah Cluster Hospitals perceived the scope of nursing practice of the staff under their oversight as variably enacted and frequently constrained. Four themes illustrate the key dimensions of these managers’ and supervisors’ scope-of-practice perceptions: the need for expanded and standardized scope definitions; the challenge of translating educational preparation into clinical practice; the systemic administrative, staffing, and resource-related barriers that limit full professional enactment; and the centrality of autonomy, accountability, and collaborative practice to nurses’ professional identity.

These findings extend and deepen the existing quantitative Saudi literature on the scope of practice, providing explanatory context for the patterns of role overlap and variable ASCOP scores documented in the pre-2023 period [4,18]. As one of the first qualitative studies conducted following the 2023 SCFHS Scope of Nursing and Midwifery Practice update, this work establishes an empirical baseline for understanding how the new national regulatory framework is being implemented and experienced by nurse managers and supervisors in clinical practice. The persistence of scope variability, administrative barriers, and training-to-practice gaps more than two years after the document’s release, as perceived by this managerial cohort, is a significant finding, suggesting that regulatory publication may be a necessary but insufficient condition for professional transformation. Because these findings reflect an organizational oversight perspective rather than the direct accounts of bedside nurses, they should be generalized with appropriate caution beyond nurse managers and supervisors in comparable cluster hospital settings. Future research should include longitudinal designs to evaluate the impact of scope-of-practice policy implementation, qualitative studies centered on bedside nursing staff, studies employing multiple independent coders and formal translation validation procedures to strengthen methodological rigor, and multi-site investigations that enable comparative analysis across health clusters and regions within Saudi Arabia.

## Figures and Tables

**Table 1:** Demographic characteristics of participants in the qualitative phase (n = 20).

Variable	Category	Frequency (n)	Percentage (%)
Gender	Female	10	50
	Male	10	50
Age (years)	Mean ± SD: 38.65 ± 3.21		
Education level	Bachelor’s degree	4	20
	Master’s degree	14	70
	PhD	2	10
Hospital	Hera General Hospital	9	45
	King Faisal Hospital	11	55
Professional role	Managerial roles (nursing directors, nursing supervisors, head nurses)	20	100

**Table 2:** Themes pertaining to the scope of nursing practice, as reported by nurse managers and supervisors.

Theme	Description
1. Expanding the scope of nursing practice	Variability in scope enactment across departments, shifts, and authority levels. Need for standardized, evidence-based scope definitions.
2. Bridging the gap between training and practice	Challenges in translating theoretical education to clinical practice. Importance of continuous professional development and simulation-based learning.
3. Overcoming barriers to full professional practice	Impact of unclear job descriptions, administrative constraints, staffing shortages, and resource deficits on scope enactment.
4. Promoting nurse empowerment and accountability	The importance of autonomy, accountability, evidence-based care, and interdisciplinary collaboration in defining and sustaining an effective scope of practice.

## Data Availability

The datasets generated during this study are available upon reasonable request by sending an email to alikariri@ksu.edu.sa due to privacy reasons.

## Supplementary Materials

The following supporting information can be downloaded at https://www.mdpi.com/article/10.3390/healthcare14142198/s1, File S1: Semi-structured interview guide.

## Abbreviations

The following abbreviations are used in this manuscript:

A-ASCOP	Arabic Actual Scope of Nursing Practice
APN	Advanced Practice Nurse
COREQ	Consolidated Criteria for Reporting Qualitative Research
IRB	Institutional Review Board
MENA	Middle East and North Africa
NMPC	Nursing and Midwifery Professional Council
QNC	Queensland Nursing Council
SCFHS	Saudi Commission for Health Specialties
SD	Standard Deviation
