# Supplementary material for: Perceptions of Nurse Managers and Supervisors on the Scope of Nursing Practice Following the 2023 National Regulatory Update in Saudi Arabia: A Qualitative Study in Makkah Cluster Hospitals
Source: Healthcare (Basel). 2026 Jul 20;14(14):2198. doi: 10.3390/healthcare14142198 (PMC13411853; doi:10.3390/healthcare14142198)
Supplement: Supplementary file 1 [file healthcare-14-02198-s001.zip › healthcare-4406186-supplementary.pdf]

SEMI-STRUCTURED INTERVIEW QUESTIONS

Study Title:

Perceptions of Nurse Managers and Supervisors on the Scope of Nursing Practice Following the 2023 National Regulatory Update in Saudi Arabia: A Qualitative Study in Makkah Cluster Hospitals

Qualitative Phase:

Qualitative Descriptive Study

Grand Tour Question:

What are the perceptions and experiences of nurse managers and supervisors working in Makkah Cluster hospitals regarding the scope of nursing practice?

Semi-Structured Interview:

Introductory:

- Could you please introduce yourself and share something about your current role, workplace, and professional life?

Scope of Nursing Practice:

- What should the scope of good nursing practice cover in a clinical setting such as yours? How does that compare with what you actually see in practice? Tell me about it.
- Thinking about the range of nursing activities — assessment and care planning, teaching patients and families, communication and coordination, supervision of staff, quality and patient safety, and updating knowledge — which do nurses in your area perform most fully, and which least? What do you think explains that?
- How are tasks and responsibilities divided between nurse specialists, nurse technicians, and other nursing staff in daily practice? Describe a situation where responsibilities overlapped or were unclear. What happened?
- How well do nurses' education and training prepare them for what their scope requires? How are their competencies assessed in your hospital?
- What helps nurses in your hospital practice to the full extent of their scope? What makes it difficult? Can you give an example of how a barrier plays out in a real situation?

Closing:

- Is there anything we have not discussed that you feel is important about the scope of nursing practice?

NOTE:

Informed consent must be obtained from participants before the interview begins. A brief explanation of the study aims and objectives must be provided. The semi-structured interview

questions will be followed by neutral probing questions (e.g., “Can you tell me more about that?”, “Can you give me an example?”, “What happened next?”) until saturation of the topic is reached. Probes are optional and used only when the content has not emerged spontaneously. The interview session will last 30–60 minutes, conducted in Arabic via Zoom, depending on the flow of the conversation between the participant and the interviewer.
